# Dietary fibre supplementation in late gestation modulates gut microbiota and improves reproductive performance and colostrum quality in sows

**DOI:** 10.3389/fmicb.2025.1667697

**Published:** 2025-12-04

**Authors:** Guanglei Cong, Chunxue Liu, Shuangshuang Xia, Junbo Li, Ifen Hung

**Affiliations:** 1 Anyou Biotechnology Group Co., Ltd., Taicang, Jiangsu, China; 2 College of Animal Science and Technology, Nanjing Agriculture University, Nanjing, Jiangsu, China

**Keywords:** fibre, reproductive performance, colostrum quality, constipation, intestinal flora, eNOS, piglet, correlation

## Abstract

**Introduction:**

Dietary fibre in late gestation may affect sow physiology, colostrum quality, gut microbiota, and piglet performance. This study examined whether increasing fibre levels enhances sow health and reproductive outcomes.

**Methods:**

Forty pregnant Landrace × Yorkshire sows (parity 2 or 4) were allocated to either a low-fibre (LF, 5.2%) or high-fibre (HF, 7.7%) diet from day 85 of gestation until parturition, after which all sows received a common lactation diet. Sow performance, constipation, nutrient digestibility, colostrum quality, cord blood parameters, and gut microbiota profiles were assessed. Piglet growth performance was recorded until weaning. Correlation analyses were conducted to examine associations between bacterial taxa and physiological indicators.

**Results:**

HF sows showed improved piglet vitality (*p* < 0.05), higher faecal scores, and lower constipation rates (*p* < 0.05). Colostrum protein, total solids, IgA, and IgM concentrations were increased in HF sows (*p* < 0.05). Cord blood glucose and IL-10 were reduced, whereas eNOS was elevated (*p* < 0.05). Digestibility of crude fibre, ADF, and NDF increased, while protein digestibility decreased (*p* < 0.05). Gut microbiota analysis indicated increased abundances of *Lactobacillus*, *Lachnospiraceae*_*XPB*1014_*group*, and *Methanobrevibacter*, and decreased *Treponema*, *Prevotellaceae*_*UCG*-001, and *Escherichia*-*Shigella*. Piglets from HF sows exhibited greater average daily gain and weaning weight (*p* < 0.05). Beneficial associations were observed for *Lactobacillus*, *Terrisporobacter*, and *Lachnospiraceae*_*XPB*1014_*group* with reproductive traits; *Phascolarctobacterium* and *X*-*Eubacterium*_*ruminantium*_*group* correlated with piglet growth; while *Treponema* and *Rikenellaceae*_*RC*9_*gut*_*group* were negatively associated with constipation.

**Discussion:**

Higher dietary fibre in late gestation improved gastrointestinal function, colostrum quality, and piglet growth, likely through enhanced fibre digestibility and favourable modulation of gut microbiota. These findings support increased fibre supplementation to optimise sow and piglet health.

## 1 Introduction

Constipation in sows is a prevalent issue influenced by physiological and external factors (restrictive stall structures, low exercise, insufficient dietary fibre content, etc.), particularly during late pregnancy (Yu et al., 2021). This condition can disrupt intestinal microflora (Ohkusa et al., 2019), reducing feed intake, impairing digestion and nutrient absorption, and increasing systemic toxin levels (Iancu et al., 2023). In pregnant sows, constipation can adversely affect embryonic development, prolong labour, and result in weaker piglets. For lactating sows, constipation may cause insufficient or absent milk production post-partum, diminish milk quality, and potentially lead to secondary conditions such as metritis and mastitis. These issues negatively impact piglet survival, growth, and development, compromising overall pig production efficiency (Pearodwong et al., 2016; Ma et al., 2023).

Fibre, often called the “seventh” nutrient, promotes intestinal peristalsis and alleviates constipation (Jørgensen et al., 2010). Soybean hulls, a by-product of soybean processing, are rich in fibre and pectin while containing minimal starch (Colletti et al., 2020). With a moisture content of about 10%, soybean hulls have 80% dry matter as carbohydrates, including approximately 20% non-fibre carbohydrates. They are low in lignin, have good palatability, and are highly digestible (Liu and Li, 2017). For monogastric animals, soybean hulls are an excellent fibre source due to their high cellulose and hemicellulose content, which ferments into volatile fatty acids in the cecum, providing energy. Additionally, soybean hulls are cost-effective and abundant, reducing feed costs and enhancing pig breeding efficiency (Goehring et al., 2019). In China, soybean hulls can replace roughage and some grain feeds like corn and wheat more cost-effectively. Adding 21% soybean hulls to the diet of pregnant sows can increase feed intake and reduce body loss during lactation (Renteria-Flores et al., 2008).

Loisel et al. (2013) incorporated soybean hulls, sugar beet pulp (SBP), wheat bran, and unshucked sunflower seed powder into the base diet, increasing the crude fibre (CF) level to 7.9%. They observed significant improvements in sow production performance and a 29% increase in the milk fat content of colostrum. However, due to variations in fibre sources, composition, dosage, diet, and sow physiology, fibre can have differing effects on sow reproductive performance (Li et al., 2021; Shi et al., 2021). Currently, there is no consensus on the impact of fibre on sow reproductive performance, and further research is needed to elucidate the effects of fibre on colostrum quality and intestinal flora. Therefore, this study was conducted to evaluate the impact of increased dietary fibre levels during late pregnancy on sow reproductive performance, colostrum quality, blood biochemistry, constipation, immune levels, intestinal flora, and the growth performance of suckling piglets, and to analyse the correlation between phenotypes and intestinal flora.

## 2 Materials and methods

The experiment was conducted at the swine experimental unit of Anyou Biotechnology Group Co., Ltd. (Nanjing, China). All experimental procedures were approved by the Committee on Ethics in the Use of Animals (CEUA) of the Anyou Biological Technology Group Co., LTD (No: ANS-CEUA- RD/PJT/PL/202310/104).

### 2.1 Experimental design, diets, and management

Forty healthy pregnant sows (Landrace × Yorkshire, parity 2 or 4, with similar farrowing dates) were randomly assigned to two dietary groups, each consisting of 20 replicates (one sow per replicate). From 30 days before farrowing until the day of parturition, the LF group was fed a diet with 5.2% crude fibre (CF), while the HF group received a diet with 7.7% CF. The 2.5% difference in CF was achieved mainly by increasing the contents of neutral detergent fibre (NDF) from 16.8 to 20.3% and acid detergent fibre (ADF) from 5.6 to 8.5% in the HF diet, through adjustments of wheat bran, corn bran, and soybean hulls, while other nutrients were kept comparable between diets (Table 1). Following parturition, all sows were given a uniform lactation diet. The study lasted 51 days, from 30 days before farrowing to 21 days post-weaning. Detailed composition and nutrient levels of the diets are outlined in Table 1.

**Table 1:** Ingredients and chemical composition of diet (air-dried basis).

Composition, %	Late pregnancy		Lactation
	LF group	HF group	
Corn	31.51	26.48	39.40
Hulled barley	15.00	15.00	–
Hull-less barley	–	–	15.00
DDGS	7.50	7.50	5.00
Wheat middlings	8.00	8.00	–
Wheat Bran	15.00	15.00	10.62
Full-fat rice bran	11.50	11.50	6.50
Soybean hulls	0.70	8.50	–
Soybean meal, 46%	5.00	2.00	14.50
Fish meal, 67% CP	–	–	1.00
Limestone	1.14	1.04	1.08
CaHPO_4_	0.38	0.44	0.65
NaHCO_3_	0.12	–	–
Soya-bean oil	1.15	1.54	2.25
Premix^a^	3.00	3.00	4.00
Total	100	100	100
Chemical composition ^b^			
NE, kcal/kg	2,300	2,300	3,000
Crude protein, %	14.00	13.85	17.78
Crude fat, %	5.80	6.20	6.40
Crude fibre, %	5.20	7.70	4.00
NDF, %	16.80	20.30	12.60
ADF, %	5.60	8.50	4.20
Crude ash	5.40	5.30	5.40
TP, %	0.70	0.73	0.63
Ca, %	0.71	0.74	0.87
Lys, %	0.90	0.97	1.19
Met, %	0.16	0.15	0.21

^a^Premix provided per kilogram of complete diet: vitamin A, 13, 000 IU; vitamin D3, 2020 IU; vitamin E, 40 mg; vitamin K3, 3.0 mg; vitamin B1, 3 mg; vitamin B2, 3.5 mg; vitamin B6, 2.5 mg; vitamin B12, 0.04 mg; niacin, 30 mg; vitamin C, 300 mg; folic acid, 1.5 mg; biotin, 0.3 mg; Fe (FeSO4·H2O), 100 mg; Cu (CuSO4·5H2O), 12 mg; I (KI), 0.3 mg; Se (Na2SeO3),0.2 mg; Zn (ZnSO4·H2O), 40 mg; Mn (MnSO4·H2O), 10 mg. ^b^NE is calculated, and other nutrient levels are measured values.

At farrowing (defined as day 0 of lactation), the numbers of live piglets, stillborn piglets (including deformed piglets), and mummified fetuses were recorded. Individual birth weights of live piglets were measured. Considering similar total numbers of live piglets per treatment, no cross-fostering was performed. Within 3 days after birth, piglets received an intramuscular iron injection, and routine procedures including tail docking, tooth clipping, and castration were conducted on day 3. Piglets were housed in incubators maintained at 22–32 °C, supplemented with heating lamps.

Sows were housed in environmentally controlled pens with slatted floors, ad libitum access to water via duckbill drinkers, and automatic feeders. The ambient temperature of the sow room was maintained at 20–24 °C with 60–70% relative humidity. During lactation, feed was provided at 07:30, 13:30, and 19:30 h, starting at 1 kg per sow on day 1 and gradually increasing by 1 kg per day until day 6. Thereafter, sows had free access to feed until day 21. The daily feed intake of each sow was recorded.

### 2.2 Measurement

The measurement of back fat (BF) thickness and BW of unfed sows was carried out on the 85th prenatal day, and the 1st and 21st postnatal days. The BF thickness was measured at the left side dorsal midline (distance 65 mm) of the 10th rib with ultrasound (Shu Shuang Lean-Meater, China).

To calculate the average weight of piglets per litter and average weight of piglets born alive, the weight of each piglet was recorded during delivery (before eating colostrum). The number of piglets per litter (including total born, born alive, healthy piglets, Weak piglets and mummified and stillborn foetus) was recorded. Healthy piglets refer to piglets with birth weight ≥ 0.8 kg, and weak piglets refer to piglets with birth weight < 0.8 kg (according to farm management standards). The duration of farrowing, defined as the time from the birth of the first piglet to the last piglet in the litter, was recorded. All observations were conducted by the same trained technician to ensure consistency and accuracy of data collection. The daily feed intake of each sow was recorded to calculate the total feed intake and average daily feed intake.

To evaluate litter uniformity, the standard deviation (Litter BW-SD) and coefficient of variation (Litter BW-CV) of piglet birth weight within each litter were calculated. Litter BW-SD was computed as the standard deviation of individual piglet birth weights within the litter, and Litter BW-CV was calculated as (Litter BW-SD / Litter BW mean) × 100%. These indicators were used to assess the uniformity of piglet birth weights.

Visual assessment of the piglet vitality scale (VS) was performed immediately after birth according to Baxter et al. (2012). Intrauterine growth retardation (IUGR) was also measured after birth and the IUGR rate was calculated.

On 5 and 21-day lactation, the BW of individual piglets was weighed. During the postnatal period, piglet mortality and diarrhoea were recorded daily. At the same time, feed intake of sows was recorded from day 85 gestation to day 21 lactation.

### 2.3 Sample collection

At parturition of delivery, 6 sows were randomly selected to collect umbilical vein blood (6 umbilical veins were randomly selected in each sow). Serum samples were obtained by centrifuging the blood at 2146 × g and 4 °C for 15 min and then stored at −80 °C until analysis. About 20 mL of colostrum was collected from the third and fourth pairs of hole heads on one side of the sow at 8 h after delivery, gently mixed and stored at −20 °C until analysis.

#### 2.3.1 Determination of routine blood level of umbilical cord blood

The levels of white blood cell (WBC), Lymphocyte (LYM), Red blood cell (RBC), Hemoglobin (HGB), hematocrit (HCT) and platelet (PLT) in sow umbilical cord blood were determined by BH-5160 Vet animal five-classification automatic haematology analyser (URIT, China).

#### 2.3.2 Analysis of the contents of hormones and metabolites in umbilical cord blood

The contents of prolactin (PRL) and Endothelial nitric oxide synthase (eNOS) were analysed using the respective enzyme-linked immunosorbent assay (ELISA) kits (Jiangsu Meimian Industrial Co., Ltd., China) following the manufacturer’s instructions and the serum concentrations of glucose (GLU), calcium (CA), triglyceride (TG), total cholesterol (TC), High-density lipoprotein cholesterol (HDL-C) and low-density lipoprotein cholesterol (LDL-C) were determined using Hitachi Automatic Biochemical Analyser 3,100 (Hitachi Diagnostic Products Co., Ltd., China). The minimal detection limits for PRL and eNOS were 8 ng/L and 0.1 μmol/L, respectively, and the intra-assay coefficient of variation (CV) of all kits was 10%, and the inter-assay CV was 12%. These detection limits are appropriate for sow serum samples and sufficient to accurately quantify physiological concentrations of PRL and eNOS in this study.

#### 2.3.3 Analysis of the content of components in colostrum and determination of milk yield

Thawed colostrum samples were analysed using MilkoSca FT3 milk analyser (FOSS, Denmark) to assess the fat, protein, and lactose contents. The results were calculated as percentages of colostrum and milk. Milk yield during lactation was evaluated based on the average daily gain (ADG) of piglets and the number of litters, using the following formula: milk yield = piglet ADG × number of piglets per litter × days of lactation × 4 (Bolduan et al., 1990). From this, it concluded the average daily milk production of sows.

#### 2.3.4 Analysis of immunoglobulin content in colostrum

The contents of immunoglobulin A (IgA), IgG and IgM were analysed using the respective enzyme-linked immunosorbent assay (ELISA) kits (Jiangsu Meimian Industrial Co., Ltd., China) following the manufacturer’s instructions. The lowest detectable levels of kit IgA, IgG and IgM were 1 μg/mL, 1.2 μg/mL and 12 μg/mL respectively, and the intra-assay coefficient of variation (CV) of all kits was 10%, and the inter-assay CV was 12%. The ELISA kits were validated for porcine colostrum by the manufacturer, and their accuracy and reproducibility were confirmed before sample analysis.

#### 2.3.5 Analysis of amino acid content in colostrum

According to the method proposed by Nascimento et al. (2020), appropriate samples were transferred to a 50 mL hydrolysis tube, 20 mL of 6 moL/L HCL was added, and then hydrolyzed at 110 °C for 24 h in an electric blast drying oven. Remove and cool, transfer to 25 mL colourimetric tube constant volume.

Accurately take 100 μL sample in 15 mL centrifuge tube, put it in a vacuum drying oven, dry it for 2 h at 60 °C (dry all solvents), fill the centrifuge tube with nitrogen, and accurately add 50 μL derived reagents: Ethanol: phenyl isothiocyanate: Water: triethylamine = 7:1:1:1 (ready to use, filled with nitrogen when preparing), derived at room temperature for 30 min, added mobile phase A (31.815 g sodium acetate +3,880 mL water +120 mL acetonitrile), fixed volume to 0.5 mL, mixed well, over 0.45 μm organic membrane coating.

AA concentrations from colostrum was analysed using oxidation analysis method on a 1,260 Infinity II Prime LC System (Agilent, United States) equipped with an RP-C18 SHISEIDO (250 mm length, 4.6 mm diameter, 5 mm particle size).

#### 2.3.6 Analysis of immune level in umbilical cord blood

The contents of interleukin-6 (IL6) and IL-10 were analysed using the respective enzyme-linked immunosorbent assay (ELISA) kits (Jiangsu Meimian Industrial Co., Ltd., China) following the manufacturer’s instructions. The lowest detectable levels of kit IL-6 and IL-10 were 50 ng/L and 8 ng/L, respectively, and the intra-assay coefficient of variation (CV) of all kits was 10%, and the inter-assay CV was 12%.

#### 2.3.7 Fecal sensory scoring criteria

Faecal scores of sows from late gestation through lactation were recorded based on the criteria outlined in Table 2. Scores below 3 were classified as constipation, while scores of 3 or above, up to a maximum of 5, were considered normal. Fecal scoring was independently conducted by three trained evaluators at the same time following unified criteria, and scoring consistency was ensured through prior training and standardisation.

**Table 2:** Sensory scoring criteria of sow faeces.

Faecal state	Stool appearance description	Score
Very severe constipation	Undefecation	0
Severe constipation	Grainy, hard and dark	1
Moderate constipation	The stool is firm and dry	2
Near-normal stool	Slightly firm, slightly dry	3
Normal bowel movement	Soft, but firm and shaped	4
Mild diarrhoea	Shaped but not firm, somewhere between normal and diarrhoea	5
Severe diarrhoea	Diarrhoea, no fixed shape, and liquid	6

#### 2.3.8 Determination of apparent nutrient digestibility

The crude protein (CP; N × 6.25), ether extract (EE), crude fibre (CF), neutral detergent fibre (NDF), and acid detergent fibre (ADF) contents in both feed and faeces were determined according to the GB/T 6436–2018, GB/T 6434–2006, GB/T 6434–2022, GB/T 20806–2022, and NY/T 459–2022 methods, respectively. Acid-insoluble ash (AIA) was assessed using the GB/T 23742–2009 method. The apparent digestibility of CP, EE, CF, NDF, and ADF was calculated based on the AIA method.

#### 2.3.9 Analysis of intestinal flora diversity

We used a DNA Kit (DP328, Tiangen Biotechnology Co., Ltd.) to extract the total genomic DNA. The integrity and concentration of RNA were detected by NanoDrop ND 2000 (Thermo, United States). According to the target fragment, PCR amplification on the V3-V4 region of 16 S rDNA, 341 F-(5’-CCTAYGGGRBGCASCAG-3′), and 806 R-(5’-GGACTACNNGGGTATCTAAT-3′). We then used 1.5% agarose gel electrophoresis to extract PCR products of 400–450 bp fragments, purified by GeneJET Gel Extraction Kit (Thermo, United States). The library was established by Ion Plus Fragment Library Kit (Thermo, United States). After Qubit quantification and library testing, each replicate 16 S rDNA was pooled and paired-end sequenced on IonS5TMXL sequencing platforms (Novogene Biotechnology Co., Ltd., China).

Sequencing was performed using the IonS5™ XL platform. Raw tags were quality-filtered using FLASH (V1.2.7). Effective tags were then extracted. These tags were clustered into operational taxonomic units (OTUs) at 97% sequence similarity using Uparse (V7.0.1001). Taxonomic annotation was conducted with the SSUrRNA database, applying a minimum confidence threshold of 0.7. Alpha and beta diversity were analysed at the OTU level. For beta diversity, Linear Discriminant Analysis Effect Size (LEfSe) was used. The LDA score threshold was set at ≥2.0, and statistical significance was determined by the Kruskal-Wallis H test (*p* < 0.05).

### 2.4 Statistical analysis

All data were initially organised using Excel 2021 and analysed using the T-test procedure in SAS 9.4 (SAS Institute Inc., Cary, NC, United States). Each sow was regarded as one experimental unit for reproductive performance (e.g., litter size, litter birth weight, piglet survival, average daily gain, and weaning weight), because these parameters were recorded at the litter level. For biochemical indices, antioxidant parameters, and gut microbiota, data obtained from the sampled sows within each group were considered as the experimental unit. Results are presented as means ± SEM. Differences were considered statistically significant at *p* < 0.05, and 0.05 ≤ *p* < 0.10 was regarded as a trend.

## 3 Results

### 3.1 Backfat thickness and feed intake

As shown in Table 3, different fibre levels had no significant effects on initial backfat, parturient backfat, weaning backfat, backfat loss, gestational feed intake and lactation feed intake of sows (*p* > 0.05).

**Table 3:** Effects of dietary fibre levels on backfat thickness and feed intake of sows (means ± SEM, *n* = 20 per group).

Item	LF group	HF group	*p*-value
Backfat at 26 d before delivery, mm	19.750 ± 0.566	20.444 ± 0.595	0.404
Backfat at delivery, mm	20.500 ± 0.555	21.118 ± 0.629	0.465
Backfat at weaning, mm	17.350 ± 0.431	17.529 ± 0.589	0.804
Backfat loss, mm	3.150 ± 0.525	3.588 ± 0.601	0.584
ADFI of late pregnancy, kg/d	3.529 ± 0.058	3.496 ± 0.053	0.675
ADFI of during lactation, kg/d	5.942 ± 0.150	5.799 ± 0.202	0.568

### 3.2 Umbilical cord blood routine

As shown in Table 4, different fibre levels had no significant effects on white blood cell count (WBC), lymphocyte count (LYM), red blood cell count (RBC), haemoglobin concentration (HGB), haematocrit (HCT), and platelet count (PLT) in cord blood of sows (*p* > 0.05).

**Table 4:** Effects of dietary fibre levels on routine parameters of sow cord blood (means ± SEM, *n* = 6 per group).

Item	LF group	HF group	*p*-value
WBC ^1^, 10^9/L	4.885 ± 0.854	4.182 ± 0.469	0.487
LYM ^2^, 10^9/L	3.187 ± 0.801	1.653 ± 0.280	0.111
RBC ^3^, 10^12/L	4.517 ± 0.561	4.558 ± 0.470	0.956
HGB ^4^, g/L	95.833 ± 12.221	93.833 ± 9.156	0.898
HCT ^5^, %	34.783 ± 4.191	34.117 ± 3.093	0.901
PLT ^6^, 10^9/L	132.667 ± 29.041	127.167 ± 17.651	0.875

^1^WBC, white blood cell; ^2^LYM, lymphocyte; ^3^RBC, red blood cell; ^4^HGB, haemoglobin; ^5^HCT, haematocrit; ^6^PLT, platelet.

### 3.3 Reproductive performance

As shown in Table 5, the number of healthy piglets of sows in the HF group was significantly increased, and piglet vitality was increased (*p* < 0.05). Additionally, such diets tended to reduce the stillbirth rate in sows (*p* = 0.08). However, different levels of fibre did not significantly affect the total number of piglets, number of live piglets, number of mummies, number of stillbirths, piglet weight, evenness, IUGR rate, or labour duration in sows (*p* > 0.05).

**Table 5:** Effects of dietary fibre levels on reproductive performance of sows (means ± SEM, *n* = 20 per group).

Item	LF group	HF group	*p*-value
Total number of births, *n*	17.000 ± 0.597	17.389 ± 0.606	0.651
Litter size born alive, *n*	14.895 ± 0.696	16.222 ± 0.558	0.148
Healthy litter size, *n*	13.684 ± 0.671^b^	15.833 ± 0.595^a^	0.023
Number of low-birth-weight piglets, n	0.889 ± 0.332	0.389 ± 0.143	0.176
Number of stillbirths, *n*	1.000 ± 0.306	0.471 ± 0.212	0.173
Low-birth-weight piglet rate, %	5.032 ± 1.948	2.396 ± 0.912	0.229
Stillbirth rate, %	6.246 ± 1.888	2.302 ± 1.031	0.077
Average weight of healthy piglets, kg	1.497 ± 0.060	1.549 ± 0.071	0.572
Average weight of newborn piglets, kg	1.457 ± 0.068	1.532 ± 0.073	0.459
Farrowing duration, min	175.714 ± 24.654	180.833 ± 36.098	0.907
Farrowing interval, min	9.816 ± 2.234	10.347 ± 1.879	0.865
Newborn piglet vitality	2.560 ± 0.144^b^	2.968 ± 0.015^a^	0.025
IUGR rate^1^, %	17.379 ± 6.083	7.520 ± 2.245	0.164
Litter BW-SD ^2^	0.292 ± 0.021	0.312 ± 0.015	0.435
Litter BW-CV ^3^, %	20.706 ± 1.510	21.585 ± 0.962	0.640

^1^IUGR, intrauterine growth retardation; ^2^Litter BW-SD, litter birth weight standard deviation; ^3^Litter BW-CV, litter birth weight coefficient of variation. ^a,b^Means not sharing identical superscripts in the same row are significantly different (*p* < 0.05).

### 3.4 Composition and content of colostrum

As shown in Table 6, the protein percentage and total solids in the colostrum of sows in the HF group significantly increased (*p* < 0.05). There was also a tendency for the milk yield of sows over the entire period to increase (*p* = 0.075). However, different dietary fibre levels did not significantly affect the fat, lactose, and urea nitrogen concentrations in the colostrum of sows (*p* > 0.05).

**Table 6:** Effects of dietary fibre levels on the composition of sow colostrum (means ± SEM, *n* = 6 per group).

Item	LF group	HF group	*p*-value
Fat, %	4.076 ± 0.388	6.140 ± 1.132	0.123
Protein, %	16.708 ± 0.835^b^	18.908 ± 0.416^a^	0.046
Lactose, %	3.628 ± 0.173	3.228 ± 0.149	0.118
Total solid, %	25.808 ± 1.027^b^	29.680 ± 0.967^a^	0.025
Urea nitrogen, mg/dl	52.000 ± 2.962	52.800 ± 4.881	0.892
Milk yield, kg	215.968 ± 10.220	241.394 ± 9.205	0.075

^a,b^Means not sharing identical superscripts in the same row are significantly different (*p* < 0.05).

As shown in Table 7, the levels of IgA and IgM in the colostrum of sows in the HF group were significantly increased (*p* < 0.05). Dietary fibre levels had no significant effects on IgG in the colostrum of sows (*p* > 0.05).

**Table 7:** Effects of dietary fibre levels on immunoglobulin levels in sow colostrum (means ± SEM, *n* = 6 per group).

Item	LF group	HF group	*p*-value
IgA ^1^, μg/ml	40.468 ± 0.663^b^	45.592 ± 0.424^a^	<0.001
IgG ^2^, μg/ml	463.548 ± 6.371	463.117 ± 4.699	0.958
IgM ^3^, μg/ml	39.228 ± 1.041^b^	48.707 ± 0.584^a^	<0.001

^1^IgA, immunoglobulin A; ^2^IgG, immunoglobulin G; ^3^IgM, immunoglobulin M. ^a,b^Means not sharing identical superscripts in the same row are significantly different (*p* < 0.05).

As shown in Table 8, the levels of Ser, Pro, and Tyr in the colostrum of sows in the HF group tended to increase (*p* = 0.08–0.09). Dietary fibre levels had no significant effects on Arg, Lys, Asp., Glu, Gly, His, Thr, Ala, Val, Met, Ile, Leu, and Phe levels in the colostrum of sows (*p* > 0.05).

**Table 8:** Effects of dietary fibre levels on free amino acid levels in sow colostrum (means ± SEM, *n* = 6 per group).

Item	LF group	HF group	*p*-value
Asp ^1^, mg/L	10548.650 ± 516.210	11508.167 ± 419.439	0.178
Glu ^2^, mg/L	22172.000 ± 748.812	23181.833 ± 987.866	0.482
Ser ^3^, mg/L	7488.850 ± 359.089	8460.167 ± 334.821	0.080
Gly ^4^, mg/L	4597.950 ± 287.136	5229.792 ± 235.517	0.120
His ^5^, mg/L	3996.650 ± 206.841	4332.458 ± 155.679	0.219
Arg ^6^, mg/L	8773.200 ± 392.360	9731.292 ± 356.730	0.104
Thr ^7^, mg/L	7937.200 ± 432.119	8758.833 ± 320.618	0.153
Ala ^8^, mg/L	7307.400 ± 359.846	8073.542 ± 321.873	0.146
Pro ^9^, mg/L	15073.333 ± 45.083	16686.917 ± 728.465	0.078
Tyr ^10^, mg/L	7000.900 ± 361.691	7859.208 ± 291.759	0.094
Val ^11^, mg/L	10609.400 ± 534.314	11315.000 ± 260.985	0.269
Met ^12^, mg/L	1270.650 ± 185.632	1350.750 ± 124.489	0.720
Ile ^13^, mg/L	6136.188 ± 208.075	6442.833 ± 195.577	0.328
Leu ^14^, mg/L	14717.750 ± 665.499	15793.000 ± 544.141	0.238
Phe ^15^, mg/L	7192.750 ± 347.499	7844.625 ± 269.149	0.166
Lys ^16^, mg/L	10387.500 ± 566.526	11597.667 ± 391.484	0.104

^1^Asp., aspartic acid; ^2^Glu, glutamic acid; ^3^Ser, serine; ^4^Gly, glycine; ^5^His, histidine; ^6^Arg, arginine; ^7^Thr, threonine; ^8^Ala, alanine; ^9^Pro, proline; ^10^Tyr, tyrosine; ^11^Val, valine; ^12^Met, methionine; ^13^Ile, isoleucine; ^14^Leu, leucine; ^15^Phe, phenylalanine; ^16^Lys, lysine.

### 3.5 Serum metabolites, eNOS and immune performance

As shown in Table 9, the GLU level in the cord blood of sows in the HF group was significantly decreased (*p* < 0.05), and the LDL-C level in the cord blood of sows in the HF group tended to decrease (*p* = 0.08). The levels of HDL-C, TC, TG and Ca in the cord blood of sows were not significantly affected by different dietary fibre levels (*p* > 0.05).

**Table 9:** Effects of dietary fibre levels on metabolite levels in sow cord blood (means ± SEM, *n* = 6 per group).

Item	LF group	HF group	*p*-value
HDL-C ^1^, mmol/L	0.433 ± 0.045	0.498 ± 0.058	0.409
LDL-C ^2^, mmol/L	0.658 ± 0.030	0.540 ± 0.048	0.079
GLU ^3^, mmol/L	0.296 ± 0.044^a^	0.142 ± 0.001^b^	0.025
TC ^4^, mmol/L	1.150 ± 0.072	1.115 ± 0.017	0.661
TG ^5^, mmol/L	1.245 ± 0.230	1.528 ± 0.087	0.283
Ca^6^, mmol/L	4.006 ± 0.195	4.015 ± 0.223	0.978

^1^HDL-C, high-density lipoprotein cholesterol; ^2^LDL-C, low-density lipoprotein cholesterol; ^3^GLU, glucose; ^4^TC, total cholesterol; ^5^TG, triglyceride; ^6^Ca, calcium. ^a,b^Means not sharing identical superscripts in the same row significantly differ (*p* < 0.05).

As shown in Table 10, the eNOS level in the cord blood of sows in the HF group increased significantly, while the IL-10 level decreased significantly (*p* < 0.05). Dietary fibre levels had no significant effects on PRL and IL-6 concentrations in the cord blood of sows (*p* > 0.05).

**Table 10:** Effects of dietary fibre levels on hormone, immune, and endothelial nitric oxide synthase levels in sow cord blood (means ± SEM, *n* = 6 per group).

Item	LF group	HF group	*p*-value
PRL ^1^, ng/L	282.857 ± 5.838	283.825 ± 7.759	0.923
eNOS ^2^,μmol/L	4.715 ± 0.120^b^	5.322 ± 0.106^a^	0.004
IL-6 ^3^, ng/L	1082.925 ± 13.238	1083.128 ± 49.325	0.997
IL-10 ^4^, ng/L	187.948 ± 2.282^a^	178.828 ± 2.965^b^	0.043

^1^PRL, prolactin; ^2^eNOS, endothelial nitric oxide synthase; ^3^IL-6, interleukin-6; ^4^IL-10, interleukin-6. ^a,b^Means not sharing identical superscripts in the same row significantly differ (*p* < 0.05).

### 3.6 Fecal sensory score

As shown in Table 11, the faecal score of sows in the HF group significantly increased during late gestation and 3 days before delivery, while the constipation rate significantly decreased during late gestation (*p* < 0.05). This diet had no significant effect on the faecal score or constipation rate in sows during lactation (*p* > 0.05).

**Table 11:** Effects of dietary fibre levels on constipation index of sows (means ± SEM, *n* = 20 per group).

Item		LF group	HF group	*P*-value
Constipation rate ^1^	Pregnancy	26.926 ± 7.308^a^	1.205 ± 0.574^b^	0.002
	Lactation	5.947 ± 1.673	3.961 ± 1.067	0.319
Faecal sensory scoring	Pregnancy	3.616 ± 0.113^b^	3.920 ± 0.027^a^	0.036
	3 d before delivery	2.623 ± 0.365^b^	3.588 ± 0.191^a^	0.048
	Lactation	3.919 ± 0.032	3.942 ± 0.029	0.603

^1^A score < 3 is considered as constipation. ^a,b^Means not sharing identical superscripts in the same row significantly differ (*p* < 0.05).

### 3.7 Nutrient digestibility

As shown in Table 12, the apparent digestibility of CF, ADF, and NDF in sows from the HF group increased significantly (*p* < 0.05). In contrast, the apparent digestibility of CP decreased significantly (*p* < 0.05). The apparent digestibility of EE was not significantly affected by different levels of fibre (*p* > 0.05).

**Table 12:** Effects of dietary fibre levels on apparent nutrient digestibility of sows (means ± SEM, *n* = 8 per group).

Item	LF group	HF group	*p*-value
CP ^1^, %	86.498 ± 0.305^a^	83.008 ± 0.641^b^	<0.001
EE ^2^, %	83.801 ± 0.792	83.696 ± 0.540	0.911
CF ^3^, %	54.758 ± 1.683^b^	67.334 ± 0.424^a^	<0.001
NDF ^4^, %	61.890 ± 1.644^b^	66.872 ± 0.703^a^	0.014
ADF ^5^, %	52.107 ± 2.058^b^	65.533 ± 0.703^a^	<0.001

^1^CP, crude protein; ^2^EE, crude fat; ^3^CF, crude fibre; ^4^NDF, neutral detergent fibre; ^5^ADF, acid detergent fibre. ^a,b^Means not sharing identical superscripts in the same row significantly differ (*p* < 0.05).

### 3.8 Intestinal flora diversity

**Figure 1:**
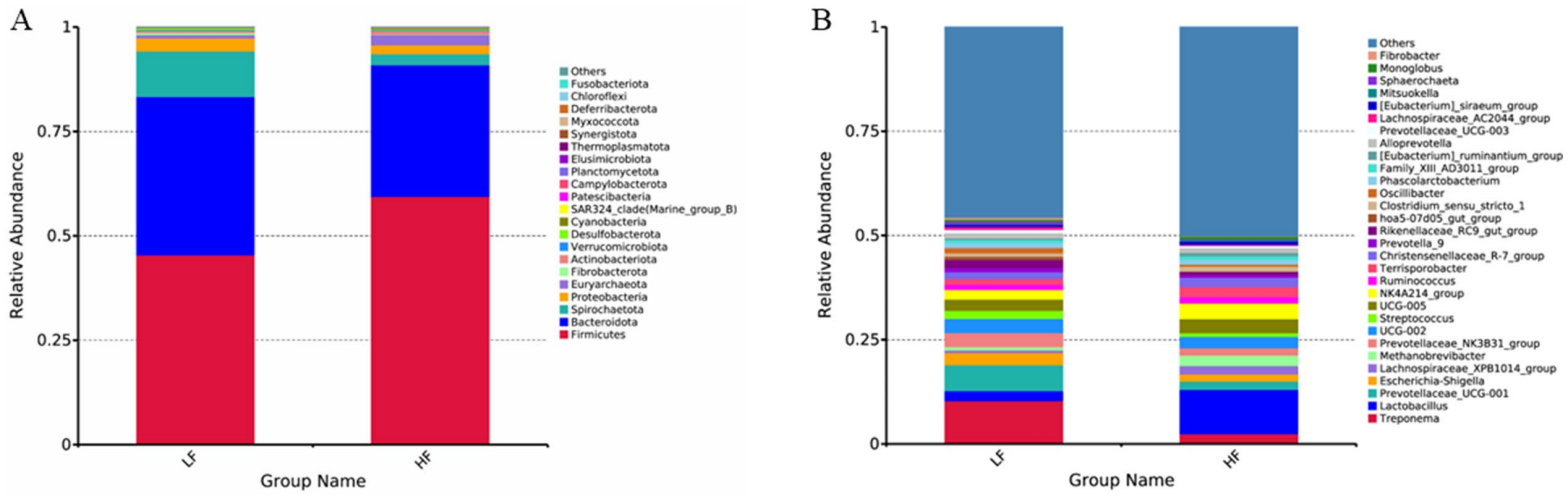
Effects of dietary fibre levels on the relative abundance of the top 21 (phylum level) and top 30 (genus level) intestinal bacterial taxa in sows (*n* = 6 per group). **(A)** Phylum level. **(B)** Genus level.

**Figure 2:**
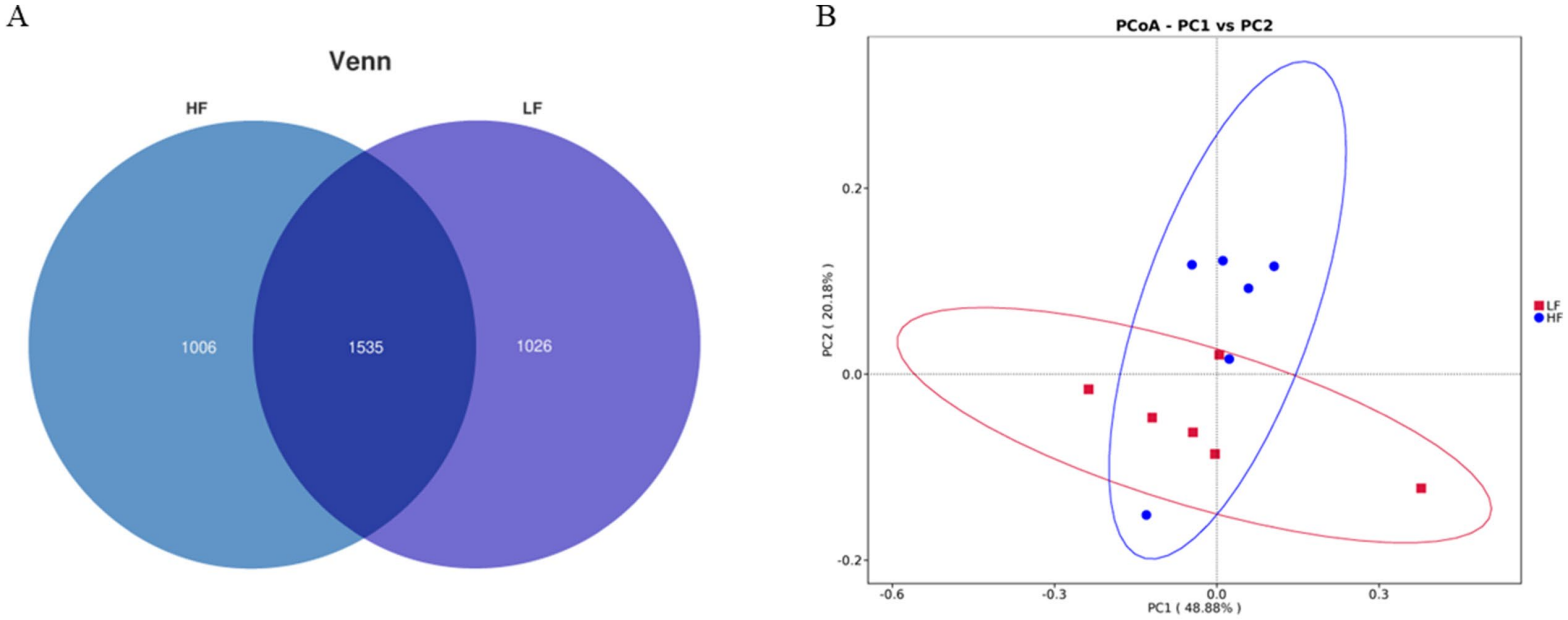
Effects of dietary fibre levels on the intestinal microbiota diversity of sows (*n* = 6 per group). **(A)** Venn diagram showing the number of shared and unique operational taxonomic units (OTUs). **(B)** Principal coordinate analysis (PCoA) based on Bray–Curtis distances.

At the phylum level, the relative abundance of Firmicutes increased in the intestinal tract of sows in the HF group, while the relative abundances of Bacteroidota, Spirochaetota, and Fibrobacterota decreased. Consequently, the Firmicutes/Bacteroidota ratio increased (1.20 vs. 1.88; Figure 1A). At the genus level, the relative abundances of *Lactobacillus*, *Lachnospiraceae*_*XPB*1014_*group*, and *Methanobrevibacte*r increased in the HF group, whereas *Treponema*, *Prevotellaceae*_*UCG*-001, and *Escherichia*-*Shigella* decreased (Figure 1B). Analysis using Venn diagrams and PCoA revealed differences in the intestinal flora of sows between the two groups (Figure 2).

**Figure 3:**
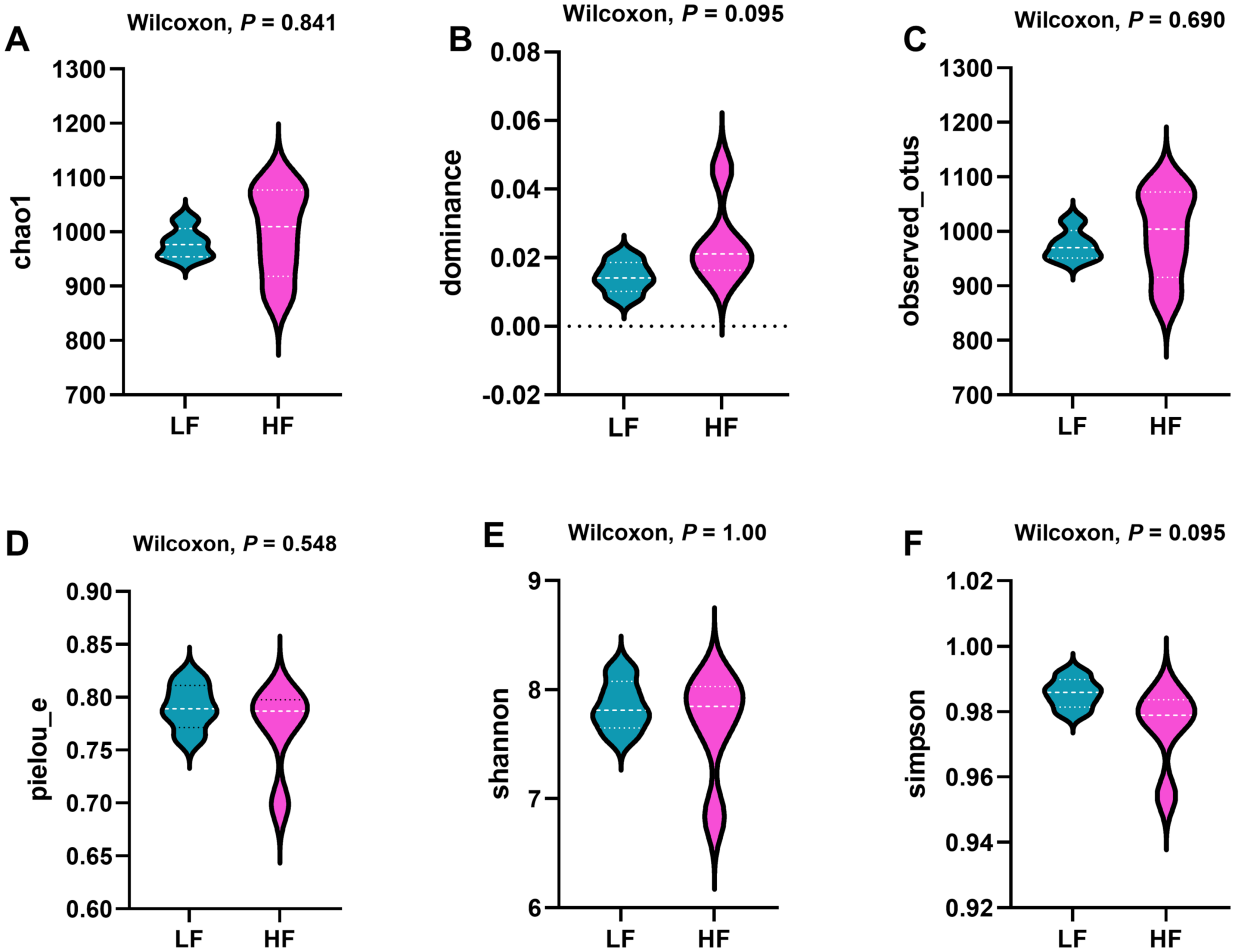
Effects of dietary fibre levels on *α* diversity of the intestinal microbiota in sows (*n* = 6 per group). **(A)** Chao1 index. **(B)** Dominance index. **(C)** Observed_otus index. **(D)** Pielou_e index. **(E)** Shannon index. **(F)** Simpson index.

Figure 3 illustrates that the dominance index of intestinal flora increased in the HF group (*p* = 0.10), while the Simpson index decreased in the LF group (*p* = 0.10). No significant effects were observed on the chao1, observed_otus, pielou_e, and Shannon indices of the intestinal flora in sows with different dietary fibre levels (*p* > 0.05).

**Figure 4:**
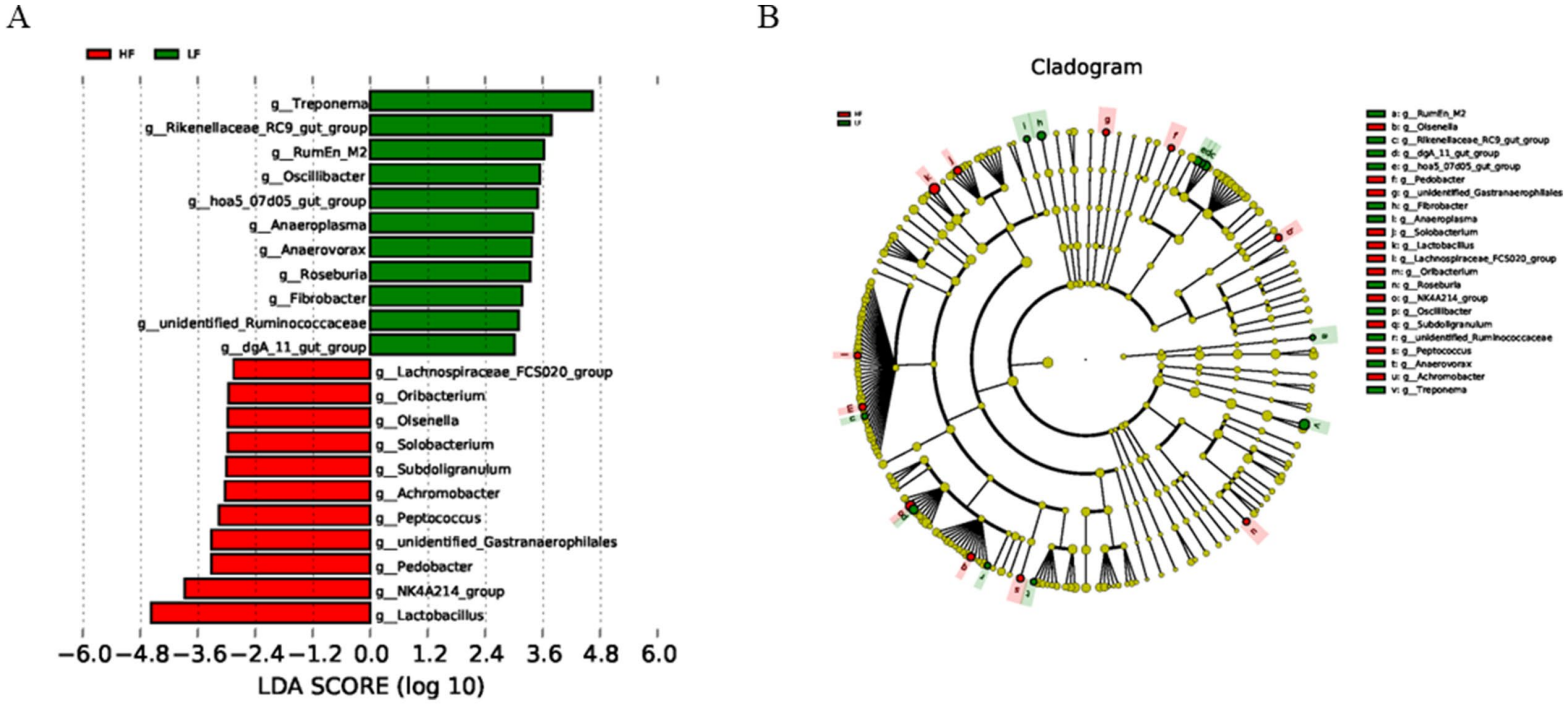
Effects of dietary fibre levels on the *β* diversity of the intestinal microbiota in sows, analysed by LEfSe (LDA score ≥ 2, *n* = 6 per group). **(A)** Linear discriminant analysis (LDA) histogram showing the distribution of discriminant taxa. **(B)** Cladogram illustrating the phylogenetic relationships among significantly different taxa.

**Figure 5:**
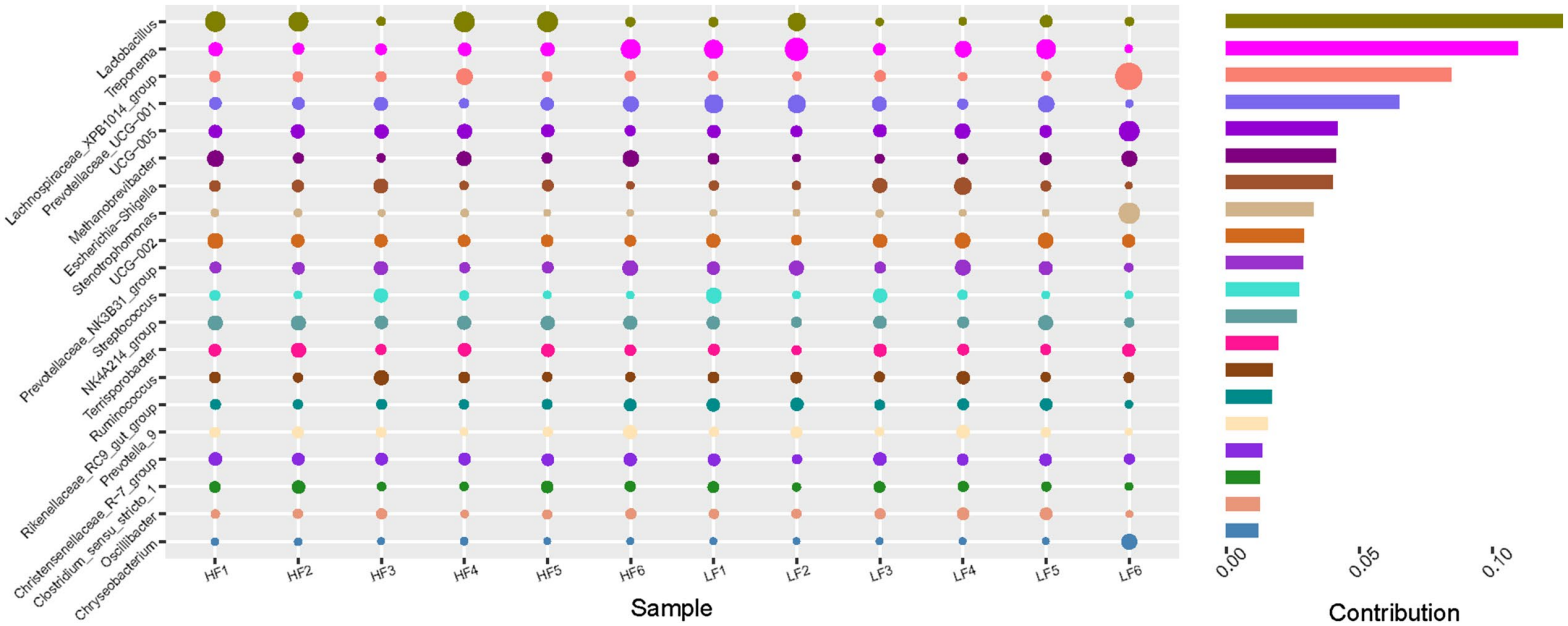
Effects of dietary fibre levels on *β* diversity of the intestinal microbiota in sows analysed by SIMPER (*n* = 6 per group).

LEfSe analysis was conducted with an LDA score threshold of ≥2.0 and Kruskal-Wallis H test *p* < 0.05. This allowed identification of taxa significantly enriched in each group. In the HF group, dominant genera included *Lactobacillus*, *NK*4*A*214_*group*, *Pedobacter*, *unidentified*_*Gastranaerophilales*, *Peptococcus*, *Achromobacter*, *Subdoligranulum*, *Solobacterium*, *Olsenella*, *Oribacterium*, and *Lachnospiraceae*_*FCS*020_group. In contrast, the LF group was dominated by *Treponema*, *Rikenellaceae*_*RC*9_*gut*_*group*, *RumEn*_*M*2, *Oscillibacter*, *hoa*5_07*d*05_*gut*_*group*, *Anaeroplasma*, *Anaerovorax*, *Roseburia*, *Fibrobacter*, *unidentified*_*Ruminoccaceae*, and *daA*_11_*gut*_*group* (Figure 4). These results indicate clear differences in gut microbiota composition between the high- and low-fibre groups. Similarity percentage analysis further confirmed that *Lactobacillus* contributed the most to the HF group, whereas *Treponema* had the highest contribution to the LF group (Figure 5).

**Figure 6:**
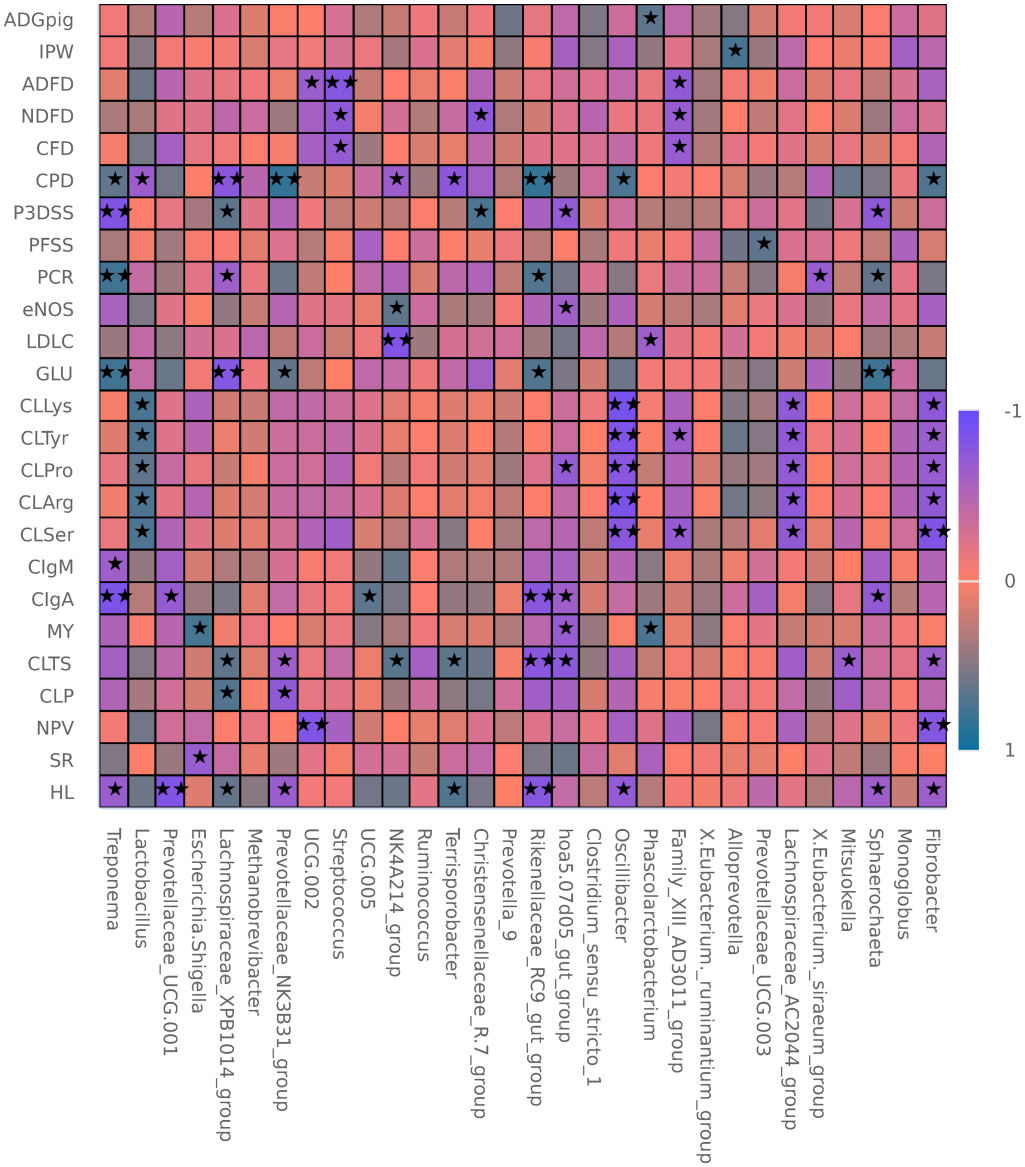
Spearman correlation analysis between sow phenotypic traits and gut microbiota under different dietary fibre levels (*n* = 6 per group). ADGpig, average daily gain of piglets; IPW, individual piglet weight; ADFD, apparent digestibility of acid detergent fibre; NDFD, apparent digestibility of neutral detergent fibre; CFD, apparent digestibility of crude fibre; CPD, apparent digestibility of crude protein; P3DSS, prepartum 3-day stool score; PFSS, pregnancy faecal sensory score; PCR, pregnancy constipation rate; eNOS, endothelial nitric oxide synthase; LDL-C, low-density lipoprotein cholesterol; GLU, glucose; CLLys, colostrum lysine; CLTyr, colostrum tyrosine; CLPro, colostrum proline; CLArg, colostrum arginine; CLSer, colostrum serine; CIgM, colostrum immunoglobulin M; CIgA, colostrum immunoglobulin A; MY, milk yield; CLTS, colostrum total solids; CLP, colostrum protein; NPV, newborn piglet vitality; SR, stillbirth rate; HL, healthy litter.

Figure 6 shows that healthy litters were positively correlated with *Terrisporobacter* and *Lachnospiraceae*_*XPB*1014_*group*, and negatively correlated with several taxa including *Oscillibacter* and *Treponema*. The stillbirth rate was negatively correlated with *Escherichia*-*Shigella*, while newborn piglet vitality was negatively correlated with *Fibrobacter* and *UCG*-002. Colostrum protein positively correlated with *Lachnospiraceae*_*XPB*1014_*group* and negatively with *Prevotellaceae*_*NK*3*B*31_*group*. Colostrum total solids positively correlated with *Terrisporobacter* and *NK*4*A*214_*group*, and negatively with several others. Milk yield was positively correlated with *Phascolarctobacterium* and *Escherichia*-*Shigella*, and negatively with *hao*-5-05*d*05_*gut*_*group*. Colostrum Immunoglobulin A positively correlated with *UCG*-005 and negatively with *hao*-5-05*d*05_*gut*_*group*. Colostrum Ser, Arg, Pro, Tyr, and Lys positively correlated with *Lactobacillus* and negatively with *Fibrobacter*. Glu and Pregnancy constipation rate positively correlated with *Treponema* and negatively with *Lachnospiraceae*_*XPB*1014_*group*. eNOS was positively correlated with *NK*4*A*214_*group* and negatively with *hao*-5-05*d*05_*gut*_*group*. Pregnancy fecal sensory scoring positively correlated with *Prevotellaceae*_*UCG*-003. Prepartum stool scores positively correlated with *Christensenellaceae*_*R*-7_*group* and negatively with *Sphaerochaeta*. CF digestibility positively correlated with *Fibrobacter* and negatively with *Terrisporobacter*. Individual piglet weight and ADG were positively correlated with *X*-*Eubacterium*_*ruminantium*_*group* and *Phascolarctobacterium*, respectively.

**Figure 7:**
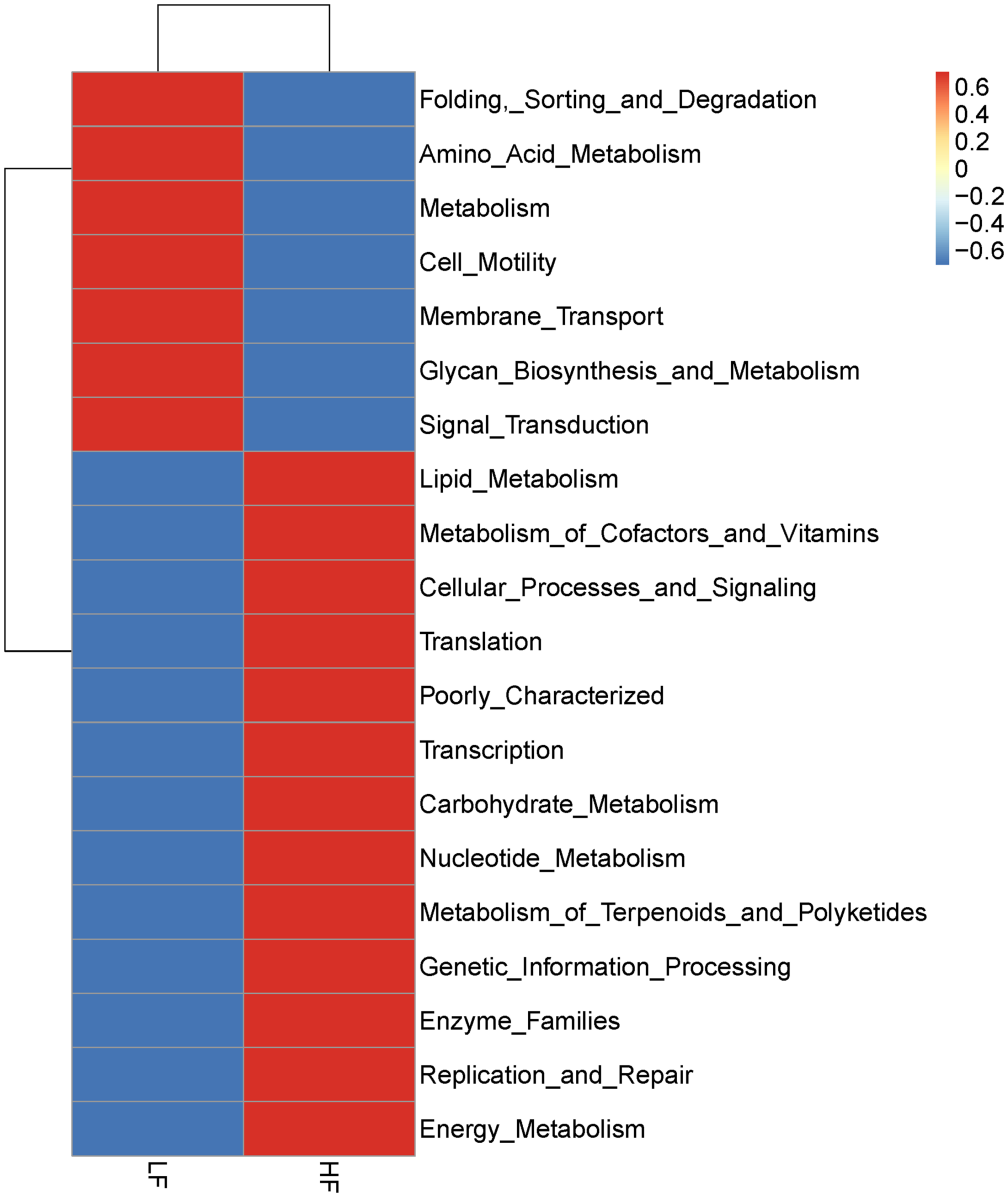
Functional prediction of the intestinal microbiota in sows fed diets with different fibre levels by PICRUSt analysis (*n* = 6 per group).

As shown in Figure 7, the intestinal flora functions of sows in the HF group were predominantly associated with carbohydrate metabolism, translation, energy metabolism, replication and repair, nucleotide metabolism, and the metabolism of cofactors and vitamins.

### 3.9 Growth performance and health of suckling piglets

As shown in Table 13, the average daily gain (ADG) and weaning weight of piglets from sows in the HF group were significantly increased (*p* < 0.05). Different fibre levels diets had no significant effects on litter size, initial litter weight, initial average weight, diarrhoea rate, or fertility rate (*p* > 0.05).

**Table 13:** Effects of dietary fibre levels on growth performance and health status of suckling piglets (means ± SEM, *n* = 20 per group).

Item	LF group	HF group	*p*-value
Litter size, n	13.500 ± 0.438	12.889 ± 0.523	0.373
Litter weight at 5 d, kg	33.670 ± 1.781	35.564 ± 2.277	0.512
Average birth weight, kg	2.414 ± 0.105	2.564 ± 0.126	0.359
Litter weight at weaning, kg	85.783 ± 3.937	90.456 ± 5.179	0.472
Individual weaning weight, kg	6.665 ± 0.176^b^	7.293 ± 0.188^a^	0.020
Average daily gain of piglets, g/d	258.631 ± 10.280^b^	286.162 ± 7.753^a^	0.043
Piglet diarrhoea rate, %	0.186 ± 0.116	0.088 ± 0.077	0.142
Survival rate, %	97.766 ± 1.844	96.448 ± 2.185	0.646

^a,b^Means not sharing identical superscripts in the same row significantly differ (*p* < 0.05).

## 4 Discussion

High-yielding sows often face challenges such as constipation and disturbances in intestinal microbiota, which can negatively affect piglet survival and maternal energy balance (Oliviero et al., 2010; Berchieri-Ronchi et al., 2011). Factors including oxidative stress and insulin resistance may exacerbate these issues, leading to reduced physical endurance during farrowing (Ju et al., 2021; Oliviero et al., 2013). In the present study, increasing dietary fibre while maintaining moderate protein levels effectively alleviated constipation, with the constipation rate reduced by approximately 25% and maintained within 1.8%. This improvement is likely attributable to fibre’s role in promoting intestinal motility, enhancing water absorption, softening faeces, and preventing excessive protein fermentation (Delimaris, 2013). Given that all sows had ad libitum access to water and environmental factors were controlled, these benefits can be primarily attributed to the dietary intervention. High-fibre diets also increased the number of healthy piglets and improved piglet vitality, whereas parameters such as total litter size, number of live-born piglets, stillbirths, litter uniformity, IUGR rate, and farrowing duration remained unchanged. This suggests that, although high-fibre diets may not alter overall litter size, they can enhance the physiological condition of sows and the viability of newborn piglets, likely through improvements in gut health, nutrient utilization, and maternal metabolic balance. The absence of significant effects on other reproductive parameters may reflect inherent biological variability or the sample size used in this study, highlighting the need for further research with larger cohorts to confirm these observations.

Constipation is often accompanied by microbial imbalance in the gut. Consistent with previous reports, increasing dietary fibre altered the microbial composition of sows in a beneficial manner (Liu et al., 2021; Zhuang et al., 2019). Specifically, the high-fibre diet increased the relative abundance of *Lactobacillus*, *Lachnospiraceae*_*XPB*1014_*group*, and *Methanobrevibacter*, while reducing potentially harmful taxa such as *Treponema*, *Prevotellaceae*_*UCG*-001, and *Escherichia*-*Shigella*. *Lactobacillus* species are well-established probiotics known to enhance intestinal barrier integrity, regulate immune responses, and inhibit enteropathogens (Wang et al., 2021; Betancur et al., 2021). The enrichment of Lactobacillus in sows fed high-fibre diets may therefore have contributed to improved intestinal health and nutrient absorption. Organic acids (e.g., lactic and acetic acid) produced by Lactobacillus lower intestinal pH, inhibit the proliferation of pathogenic bacteria such as *E. coli*(Guarner and Malagelada, 2003; Evivie et al., 2020), and modulate epithelial gene expression via lactate receptors (GPR81 and Wnt3), promoting mucosal regeneration and reducing pro-inflammatory cytokines (Averina et al., 2021). Furthermore, antioxidant enzymes and manganese ions produced by Lactobacillus can scavenge intestinal reactive oxygen species, repair oxidative damage, and maintain epithelial homeostasis, all of which contribute to improved reproductive performance. Similar findings were reported by Qin et al. (2023), Animal Feed Science and Technology, who observed that fibre-induced enrichment of Lactobacillus alleviated oxidative stress and improved farrowing outcomes in sows.

Interestingly, despite the reduction in apparent crude protein (CP) digestibility in high-fibre-fed sows, colostrum protein, total solids, IgA, and IgM concentrations significantly increased, while free amino acid levels remained unchanged. This apparent paradox can be explained by improved nitrogen utilisation efficiency. High dietary fibre has been shown to reduce urinary nitrogen excretion and promote microbial nitrogen assimilation in the hindgut, leading to more efficient amino acid recycling (Loisel et al., 2013; Shang et al., 2021; Wisbech et al., 2024). Consequently, a larger proportion of amino acids may have been channelled into colostrum protein and immunoglobulin production, even though intestinal CP digestibility declined. A similar pattern was observed in growing pigs, where fermentable fibre enhanced microbial protein synthesis and reduced plasma urea nitrogen levels (Zervas and Zijlstra, 2002). Thus, the improved colostrum composition in the present study likely reflects enhanced metabolic nitrogen retention rather than increased amino acid absorption.

*Lactobacillus* enrichment may also indirectly support improved colostrum composition by modulating lipid metabolism and bile acid homeostasis. Certain Lactobacillus strains possess bile salt hydrolase (BSH) activity, enabling them to deconjugate bile salts, lower serum cholesterol, and enhance lipid turnover (Wu et al., 2021; Dlamini et al., 2017). The observed reduction in cord blood LDL-C concentrations aligns with this mechanism, suggesting that fibre-induced microbial modulation contributed to improved lipid metabolism in sows. Lower cord blood glucose (GLU) levels may indicate enhanced insulin sensitivity, a positive metabolic adaptation during gestation (George et al., 1978). However, excessively low glucose concentrations could potentially impair fetal growth and neonatal energy supply. In this study, combining cord blood GLU measurements with newborn piglet vitality data, we found that piglet vitality remained within the normal physiological range, indicating that the reduction observed with 7.7% dietary fibre is safe and “moderately beneficial” without adverse effects.

Dietary fibre is known to influence nutrient digestibility depending on its physicochemical properties and source. Previous studies have reported that inclusion of sugar beet pulp (SBP) or alfalfa meal at 12–17% reduces apparent total tract digestibility of energy and CP, while increasing faecal N output (Le Goff et al., 2002; Krogh et al., 2015). In the present study, increasing fibre levels improved the digestibility of crude fibre (CF), neutral detergent fibre (NDF), and acid detergent fibre (ADF) by 12.6, 5.0, and 13.4%, respectively, but decreased apparent CP digestibility. This discrepancy may be due to differences in fibre type and solubility. Soluble fibres, such as pectin from beet pulp, promote microbial fermentation and short-chain fatty acid (SCFA) production, enhancing energy yield, whereas insoluble fibres mainly stimulate peristalsis and bulk formation (Slavin, 2013; Meldrum and Yakubov, 2025). As a result, the overall energy utilisation efficiency in high-fibre-fed sows may have been maintained despite reduced CP digestibility, aided by improved nitrogen recycling and microbial fermentation (Patráš et al., 2012; Yang et al., 2022).

The gut Firmicutes/Bacteroidota (F/B) ratio has been associated with anti-inflammatory effects and energy homeostasis in pigs (Hu et al., 2022). In the current study, a numerically higher F/B ratio was observed in sows fed the high-fibre diet, suggesting a shift toward microbial profiles favouring SCFA production and immune regulation. SCFAs such as acetate, propionate, and butyrate have been shown to exert anti-inflammatory and antioxidative effects by promoting regulatory T cell activity and enhancing intestinal barrier function (Park et al., 2015; Smith et al., 2013). The increased immunoglobulin concentrations (IgA and IgM) in colostrum further support the view that fibre-mediated microbial modulation enhanced maternal immune competence without perturbing endocrine parameters such as prolactin. Meanwhile, the reduction in IL-10 concentrations in cord blood may reflect a balanced immune activation state, consistent with controlled inflammation and improved metabolic stability.

Although no significant changes were detected in colostrum fat and lactose contents, the numerical increase (2%) in milk fat and protein observed in sows fed high-fibre diets indicates a potential improvement in milk quality. This may be due to increased SCFA production and enhanced nutrient absorption mediated by the Lactobacillus-dominated microbiota (Dowarah et al., 2017; Liu et al., 2019). Lactobacillus-derived metabolites have been reported to promote gluconeogenesis and lipid synthesis in mammary tissues (Dowarah et al., 2017; Li et al., 2025), which could explain the improved milk composition observed in our study.

Finally, the beneficial effects of high-fibre diets extended to offspring growth. The improved colostrum composition, together with enhanced maternal microbial and immune status, likely contributed to the observed increase in piglet average daily gain (ADG) and weaning weight. Previous studies demonstrated that dietary inclusion of beet pulp or wheat bran improved piglet growth by enhancing colostrum quality, intestinal barrier function, and reducing maternal inflammation (Shang et al., 2019). The current findings align with these reports, highlighting that moderate elevation of dietary fibre in late gestation can simultaneously optimise maternal metabolism, gut health, and neonatal performance.

In summary, high-fibre feeding in sows modulated intestinal microbiota composition, improved colostrum quality, and enhanced immune function, without compromising energy digestibility. Although apparent intestinal protein digestibility was slightly reduced, the potential effects on overall nitrogen utilisation, including urinary nitrogen excretion, remain to be investigated. These findings provide mechanistic insight into the role of dietary fibre in promoting sow health and reproductive performance, emphasising the interplay between gut microbiota, nutrient metabolism, and immune regulation during the perinatal period.

## 5 Conclusion

Increasing dietary fibre intake in late pregnancy improves intestinal health in sows by boosting beneficial *Lactobacillus*, reducing harmful bacteria, and enhancing fibre digestibility. This approach also alleviates constipation, elevates cord blood eNOS levels, and lowers LDL-C, leading to better reproductive performance, higher milk quality, and improved growth in piglet offspring.

## Data Availability

The raw data supporting the conclusions of this article will be made available by the authors, without undue reservation.
